# Multipole Expansion of the Scalar Potential on the Basis of Spherical Harmonics: Bridging the Gap Between the Inside and Outside Spaces via Solution of the Poisson Equation

**DOI:** 10.3390/ma18102344

**Published:** 2025-05-17

**Authors:** Dimosthenis Stamopoulos

**Affiliations:** Department of Physics, School of Science, National and Kapodistrian University of Athens, Zografou Panepistimioupolis, 15784 Athens, Greece; densta@phys.uoa.gr

**Keywords:** electric potential, electric polarization, magnetic pseudopotential, magnetic polarization, multipole expansion, Poisson equation, Laplace equation, variation of parameters, dielectric materials, magnetic materials

## Abstract

The multipole expansion on the basis of Spherical Harmonics is a multifaceted mathematical tool utilized in many disciplines of science and engineering. Regarding physics, in electromagnetism, the multipole expansion is exclusively focused on the scalar potential, $U\left(r\right)$, defined only in the so-called inside, $U_{in}\left(r\right)$, and outside, $U_{out}\left(r\right)$, spaces, separated by the middle space wherein the source resides, for both dielectric and magnetic materials. Intriguingly, though the middle space probably encloses more physics than the inside and outside spaces, it is never assessed in the literature, probably due to the rather complicated mathematics. Here, we investigate the middle space and introduce the multipole expansion of the scalar potential, $U_{mid}\left(r\right)$, in this, until now, unsurveyed area. This is achieved through the complementary superposition of the solutions of the inside, $U_{in}\left(r\right)$, and outside, $U_{out}\left(r\right)$, spaces when carefully adjusted at the interface of two appropriately defined subspaces of the middle space. Importantly, while the multipole expansion of $U_{in}\left(r\right)$ and $U_{out}\left(r\right)$ satisfies the Laplace equation, the expression of the middle space, $U_{mid}\left(r\right)$, introduced here satisfies the Poisson equation, as it should. Interestingly, this is mathematically proved by using the method of variation of parameters, which allows us to switch between the solution of the homogeneous Laplace equation to that of the nonhomogeneous Poisson one, thus completely bypassing the standard method in which the multipole expansion of ${\left|r-r^{\prime }\right|}^{-1}$ is used in the generalized law of Coulomb. Due to this characteristic, the notion of $U_{mid}\left(r\right)$ introduced here can be utilized on a general basis for the effective calculation of the scalar potential in spaces wherein sources reside. The proof of concept is documented for representative cases found in the literature. Though here we deal with the static and quasi-static limit of low frequencies, our concept can be easily developed to the fully dynamic case. At all instances, the exact mathematical modeling of $U_{mid}\left(r\right)$ introduced here can be very useful in applications of both homogeneous and nonhomogeneous, dielectric and magnetic materials.

## 1. Introduction

In cases where spherical coordinates are necessitated, Spherical Harmonics (SH) is a powerful series-based tool to tackle problems by tailoring flexibility, time availability, and accuracy in science and engineering. The multifaceted character of SH is obvious by the wide utilization in chemistry [1,2,3,4,5], physics [6,7,8,9], biology [10,11,12,13], earth and space sciences [14,15,16,17], engineering and applied sciences [18,19,20,21,22,23,24,25,26,27,28], and biophysics and diagnostic radiology [29,30,31,32,33,34,35], either as the solution of the Laplace equation or in the form of the multipole expansion [36,37,38]. In the field of electromagnetism, SH is routinely employed in both statics and dynamics. Most commonly, the multipole expansion of SH is used to describe the scalar field, $U\left(r\right)$, which serves as a stepping stone for the subsequent calculation of relevant vector fields. In electricity, this is the physically meaningful electric potential, $U\left(r\right)$, originating from real sources, i.e., volume density of electric charges, ρ(r). In magnetism, it is the physically meaningless, however mathematically valuable, magnetic pseudopotential, $U_{m}\left(r\right)$, associated with fictitious sources, i.e., magnetic pseudocharges, $\rho_{m}(r)$. Accordingly, from $U\left(r\right)$, we calculate the electric field, $E\left(r\right)=-\nabla U\left(r\right)$, and the accompanying polarization, $P\left(r\right)=\varepsilon_{0}\chi_{e}E\left(r\right)$, while from $U_{m}\left(r\right),$ we obtain the magnetic field, $H(r)=-\nabla U_{m}\left(r\right)$, together with the respective polarization, $M(r)=\chi_{m}H\left(r\right)$. The algebraic part of what follows focuses on the electric potential, $U\left(r\right)$.

It is a rather inexplicable fact that until today, both the theoretical investigation and utilization in applications of the multipole expansion of the electric potential, $U\left(r\right)$, has been restricted at the so-called inside, $U_{in}\left(r\right)$, and outside, $U_{out}\left(r\right)$, spaces, bordered by the middle space wherein the source resides [36,37,38]. These areas are free of any source so that the solution of the Laplace equation governs the form attained by both $U_{in}\left(r\right)$ and $U_{out}\left(r\right)$. To the best of our knowledge, the multipole expansion of the electric potential, $U\left(r\right)$, in the middle space, i.e., in the area occupied by the source, is not surveyed in the literature. Here, we investigate this issue. We introduce the multipole expansion of the scalar potential in the middle space, $U_{mid}\left(r\right)$, in a way absolutely consistent with the standard knowledge of its behavior in the inside, $U_{in}\left(r\right)$, and outside, $U_{out}\left(r\right)$, spaces. Specifically, to obtain a valid expression of $U_{mid}\left(r\right),$ we recruit the solutions of the inside, $U_{in}\left(r\right)$, and outside, $U_{out}\left(r\right)$, spaces in a complementary fashion by carefully adjusting their mathematical and physical fingerprint at the interface of two appropriately defined subspaces of the middle space. The expression of $U_{mid}\left(r\right)$ obtained under these conditions satisfies the Poisson equation, though its parent functions, $U_{in}\left(r\right)$ and $U_{out}\left(r\right)$, satisfy the Laplace equation. Interestingly, by using the method of variation of parameters (commonly employed to solve ordinary differential equations [39,40]), we prove that $U_{mid}\left(r\right)$ is obtained directly from the solutions of the Laplace equation, $U_{in}\left(r\right)$ and $U_{out}\left(r\right)$, while it satisfies the Poisson one. This is a standalone, quite interesting finding of the present work; we completely bypass the standard method in which the multipole expansion of ${\left|r-r^{\prime }\right|}^{-1}$ is used in the generalized law of Coulomb and prove on purely mathematical grounds, quite effortlessly, that the so-called multipole expansion is actually the solution of the nonhomogeneous Poisson equation. The information obtained for electricity is concisely translated to the relevant case of magnetism when pseudocharges are introduced by the presence of magnetic materials. Accordingly, the expression of $U_{mid}\left(r\right)$ introduced here is a generic and effective solver of problems referring to spaces wherein both dielectric and magnetic sources reside. We clarify that our method applies to both spatially homogeneous and nonhomogeneous sources. Finally, the concept introduced here is successfully tested against standard systems found in applications of the literature.

## 2. Background

The Laplace equation of the electric potential, $U\left(r\right)$, is the cornerstone relation of practically all boundary value problems based on separation of variables, in spaces free of any source [36,37,38]:

(1) $$\nabla^{2}U\left(r\right)=0$$

Relation (1) has the following well-known generic solution:

(2) $$U\left(r\right)=\sum_{l=0}^{\infty }\sum_{m=-l}^{l}\left(A_{l}^{m}r^{l}+B_{l}^{m}r^{-\left(l+1\right)})Y_{l}^{m}(\theta ,\varphi \right)$$

The SH, $Y_{l}^{m}(\theta ,\varphi )$, is given by the following relation [36,37,38]:

(3) $$Y_{l}^{m}\left(\theta ,\varphi \right)=\sqrt{\frac{2l+1}{4\pi }\frac{\left(l-m\right)!}{\left(l+m\right)!}}P_{l}^{m}\left(cos\theta \right)e^{im\varphi }$$

and satisfies the following normalization condition:

(4) $$\int_{0}^{2\pi }\int_{0}^{\;\pi }Y_{l^{\prime }}^{m^{\prime }}\left(\theta^{\prime },\varphi^{\prime }\right)Y_{l}^{m*}\left(\theta^{\prime },\varphi^{\prime }\right)sin\theta^{\prime }d\theta^{\prime }d\varphi^{\prime }=\delta_{ll^{\prime }}\delta_{mm^{\prime }}$$

The above expressions are preferentially used when the available boundary conditions refer to how $U\left(r\right)$ behaves at the origin of the coordinate system, at all interfaces between different media, and at infinite distance away from the origin.

**Figure 1:**
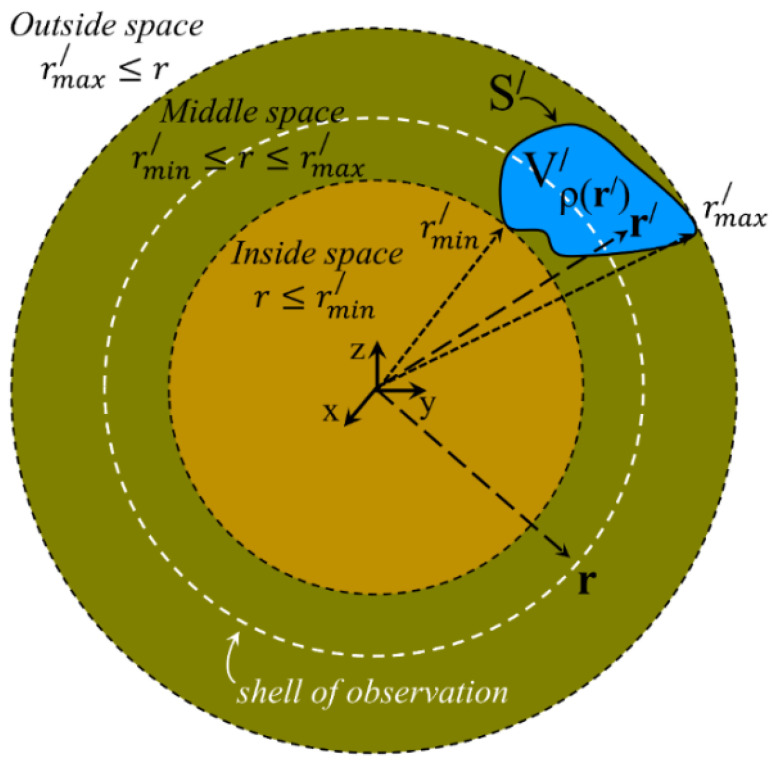
Schematic illustration of the case where a volume density, $\rho (r^{\prime })$, of free and/or bound charge, resides within a well-defined space of volume $V^{\prime }$ confined by a surface $S^{\prime }$. Two spherical shells delimit three main spaces with respect to the volume density, $\rho (r^{\prime })$. The inside space is characterized by $r\leq r_{min}^{\prime }$, where $r_{min}^{\prime }$ refers to the point of the volume density placed closest to the origin of the coordinate system. The outside space is defined through $r_{max}^{\prime }\leq r$, where now $r_{max}^{\prime }$ refers to the point of the volume density placed farthest from the origin of the coordinate system. Finally, the middle space, placed between the inside and outside ones, is determined via $r_{min}^{\prime }\leq r\leq r_{max}^{\prime }$. The ‘shell of observation’ resides in the middle space and defines the interface where the two components of the respective multipole expansion of $U\left(r\right)$ should be carefully adjusted to obtain physically meaningful results (see text for details).

On the contrary, when information on the charge density is available, the multipole expansion of $U\left(r\right)$ has obvious algebraic advantages and is definitely preferred to handle problems where, in the most general case, a volume charge density, $\rho (r^{\prime })$, free and/or bound, occupies a well-defined space of volume $V^{\prime }$ confined by a surface $S^{\prime }$. The situation is shown in Figure 1. Two spherical shells of radius $r=r_{min}^{\prime }$, the inner, and $r=r_{max}^{\prime }$, the outer, configure three main spaces in respect to the volume density, $\rho (r^{\prime })$. The inside one is characterized by $r\leq r_{min}^{\prime }$, the outside one is defined through $r_{max}^{\prime }\leq r$, and the middle space, placed between the inside and outside ones, is determined via $r_{min}^{\prime }\leq r\leq r_{max}^{\prime }$.

Once we have defined the physical system, we can now deal with the multipole expansion of $U\left(r\right)$ [36,37,38]. The concept is rather simple. The cornerstone law of Coulomb is recruited in the following generalized form [36,37,38]:

(5) $$U\left(r\right)=\frac{1}{4\pi \varepsilon_{0}}\int_{V^{\prime }}\frac{\rho (r^{\prime })}{\left|r-r^{\prime }\right|}dV^{\prime }$$

where $r^{\prime }$ runs over the volume, $V^{\prime }$, of $\rho (r^{\prime })$, while r runs over the observation points where the scalar potential, $U\left(r\right)$, should be determined. Then, based on the addition theorem, the following expansion of ${\left|r-r^{\prime }\right|}^{-1}$ is employed on the basis of SH for the inside/outside space $\left(r<r_{min}^{\prime }/r_{max}^{\prime }<r\right)$ through the following relation [36,37,38]:

(6) $$\frac{1}{\left|r-r^{\prime }\right|}=4\pi \sum_{l=0}^{\infty }\sum_{m=-l}^{l}\frac{1}{2l+1}\frac{{r_{<}^{†}}^{l}}{{r_{>}^{†}}^{l+1}}Y_{l}^{m}(\theta ,\varphi )Y_{l}^{m*}\left(\theta^{\prime },\varphi^{\prime }\right)$$

where ${r_{<}^{†}}^{l}$ and ${r_{>}^{†}}^{l+1}$ denote the lower and higher, in magnitude, between the two vectors, r and $r^{\prime }$. Thus, for the inside space $\left(r<r_{min}^{\prime }\right)$, ${r_{<}^{†}}^{l}=r^{l}$ and ${r_{>}^{†}}^{l+1}={r^{\prime }}^{l+1}$, while for the outside space $\left(r_{max}^{\prime }<r\right)$, ${r_{<}^{†}}^{l}={r^{\prime }}^{l}$ and ${r_{>}^{†}}^{l+1}=r^{l+1}$.

By using the above relations (5) and (6), we obtain for the inside space $\left(r<r_{min}^{\prime }\right)$ the following:

(7) $$U_{in}\left(r\right)=\frac{1}{4\pi \varepsilon_{0}}\sum_{l=0}^{\infty }\frac{4\pi }{2l+1}r^{l}\sum_{m=-l}^{l}q_{l,in}^{m}Y_{l}^{m}(\theta ,\varphi )$$

where $q_{l,in}^{m}$ refers to the respective multipole moments defined through the following relation:

(8) $$q_{l,in}^{m}=\int_{V^{\prime }}\rho (r^{\prime })\frac{Y_{l}^{m*}\left(\theta^{\prime },\varphi^{\prime }\right)}{{r^{\prime }}^{l+1}}dV^{\prime }$$

Also, for the outside space $\left(r_{max}^{\prime }<r\right)$

(9) $$U_{out}\left(r\right)=\frac{1}{4\pi \varepsilon_{0}}\sum_{l=0}^{\infty }\frac{4\pi }{2l+1}\frac{1}{r^{l+1}}\sum_{m=-l}^{l}q_{l,out}^{m}Y_{l}^{m}(\theta ,\varphi )$$

where $q_{l,out}^{m}$ refers to the relevant multipole moments defined through the following relation:

(10) $$q_{l,out}^{m}=\int_{V^{\prime }}\rho (r^{\prime }){r^{\prime }}^{l}Y_{l}^{m*}\left(\theta^{\prime },\varphi^{\prime }\right)dV^{\prime }$$

The above expressions of the multipole expansion of $U\left(r\right)$ at the inside space $\left(r<r_{min}^{\prime }\right)$, relations (7) and (8), and outside space $\left(r_{max}^{\prime }<r\right)$, relations (9) and (10), are routinely reproduced in the literature. On the contrary, to the best of our knowledge, there is no textbook or research article to describe the form of the multipole expansion of $U\left(r\right)$ in the middle space $\left(r_{min}^{\prime }\leq r\leq r_{max}^{\prime }\right)$. This is probably due to the demanding nature of the necessary mathematics, further complicated by the engaged underlying physics. This issue is investigated below.

## 3. Multipole Expansion of the Scalar Potential in the Middle Space

**Figure 2:**
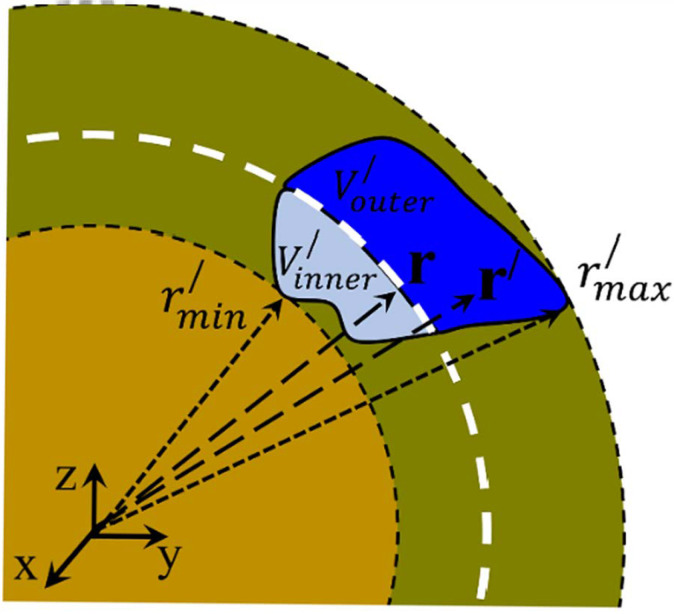
Detailed schematic illustration of how the ‘shell of observation’ (white thick-dashed curve) is defined inside the middle space ($r_{min}^{\prime }\leq r\leq r_{max}^{\prime }$). It is a thickless shell of radius r, which divides the former volume $V^{\prime }$ of the charge density, $\rho (r^{\prime })$ (see Figure 1), into two distinct subvolumes, $V_{inner}^{\prime }$ (light blue) and $V_{outer}^{\prime }$ (dark blue). Both are enclosed by the former surface $S^{\prime }$ and the ‘shell of observation’ (see Figure 1); however, the inner subvolume, $V_{inner}^{\prime }$ (light blue) spans from $r_{min}^{\prime }$ to r, while the outer subvolume, $V_{outer}^{\prime }$ (dark blue) extends from r to $r_{max}^{\prime }$.

The desired expression of the multipole expansion of the scalar potential at the middle space, $U_{mid}\left(r\right)$, should possess specific characteristics. First, $U_{mid}\left(r\right)$ should satisfy the continuity at the two spherical interfaces, that is, $U_{mid}\left(r\right)=U_{in}\left(r\right)$ at $r=r_{min}^{\prime }$ and $U_{mid}\left(r\right)=U_{out}\left(r\right)$ at $r=r_{max}^{\prime }$. Second, $U_{mid}\left(r\right)$ should satisfy the Poisson equation within the volume, $V^{\prime }$. To assess the latter issue, we introduce a spherical shell, termed ‘shell of observation’, preliminary illustrated in Figure 1. This shell is thickless, has radius r, and is placed exclusively inside the middle space ($r_{min}^{\prime }\leq r\leq r_{max}^{\prime }$). The ‘shell of observation’ divides the original volume $V^{\prime }$ of the charge density, $\rho (r^{\prime })$, in two subvolumes, the inner one, $V_{inner}^{\prime }$, which spans within $r_{min}^{\prime }$ and r, and the outer one, $V_{outer}^{\prime }$, which extends within r and $r_{max}^{\prime }$ (obviously, $V^{\prime }=V_{outer}^{\prime }\cup V_{inner}^{\prime }$). Understanding this configuration is crucial since our physical argumentation and the accompanying algebraic maneuvers are based on its clarity. This is why the configuration of the system is illustrated in detail in Figure 2.

The ‘shell of observation’ is the cornerstone of our physical argumentation. It is a spherical surface (of negligible thickness/volume) considered to be free of any charge, i.e., vacuum, that traces the radial direction from $r_{min}^{\prime }$ to $r_{max}^{\prime }$. Thus, it defines the interface that divides the former volume $V^{\prime }$ of the charge density, $\rho (r^{\prime })$, into two subvolumes, $V_{inner}^{\prime }$ and $V_{outer}^{\prime }$, of charge density $\rho_{inner}\left(r^{\prime }\right)=\rho_{r^{\prime }<r}\left(r^{\prime }\right)$ and $\rho_{outer}\left(r^{\prime }\right)=\rho_{r<r^{\prime }}\left(r^{\prime }\right)$, respectively. The spherical form of the ‘shell of observation’ is necessitated by the obvious compatibility with the expansion on the basis of SH employed in our approach. Accordingly, we can see that the calculation of $U\left(r\right)$ over the ‘shell of observation’ inside $V^{\prime }$ can be obtained by taking into account the two distinct charge densities of subvolumes $V_{inner}^{\prime }$ and $V_{outer}^{\prime }$ (see Figure 2) through the appropriate expression of the multipole expansion. Thus, the scalar potential produced on the ‘shell of observation’ by the charge density, $\rho_{inner}\left(r^{\prime }\right)=\rho_{r^{\prime }<r}\left(r^{\prime }\right)$, residing in $V_{inner}^{\prime }$ should be calculated through relations (9)–(10) of the outside space. Similarly, the scalar potential produced on the ‘shell of observation’ by the charge density, $\rho_{outer}\left(r^{\prime }\right)=\rho_{r<r^{\prime }}\left(r^{\prime }\right)$, hosted by $V_{outer}^{\prime }$ should be calculated through relations (7) and (8) of the inside space. However, a crucial point must be clarified here. While in relations (7) and (9) of $U_{in}\left(r\right)$ and $U_{out}\left(r\right)$, respectively, the radius, r, of the ‘shell of observation’ is clearly identified in relations (8) and (10) of the respective multipole moments, $q_{l,in}^{m}$ and $q_{l,out}^{m}$, the radius, r, is somehow obscured. The crucial point is to realize that in the calculation of $q_{l,in}^{m}$ and $q_{l,out}^{m}$, the radial integration should run within r and $r_{max}^{\prime }$ in relation (8) and within $r_{min}^{\prime }$ and r in relation (10). This means that the multipole moments, $q_{l,in}^{m}$ and $q_{l,out}^{m}$, are no longer constants, they have become functions of the radius, r, of the ‘shell of observation’. Thus, to calculate the scalar potential of the middle space, $U_{mid}\left(r\right)$, we first must redefine the multipole moments through the following relations:

(11) $$q_{l,in}^{m}(r)=\int_{r}^{r_{max}^{\prime }}\frac{1}{{r^{\prime }}^{l-1}}\left(\int_{0}^{\;2\pi }\int_{0}^{\pi }\rho \left(r^{\prime }\right)Y_{l}^{m*}\left(\theta^{\prime },\varphi^{\prime }\right)sin\theta^{\prime }d\theta^{\prime }d\varphi^{\prime }\right)dr^{\prime }$$

and

(12) $$q_{l,out}^{m}(r)=\int_{r_{min}^{\prime }}^{r}{r^{\prime }}^{l+2}\left(\int_{0}^{\;2\pi }\int_{0}^{\pi }\rho \left(r^{\prime }\right)Y_{l}^{m*}\left(\theta^{\prime },\varphi^{\prime }\right)sin\theta^{\prime }d\theta^{\prime }d\varphi^{\prime }\right)dr^{\prime }$$

In relations (11) and (12), the integration over the angles $\theta^{\prime }$ and $\varphi^{\prime }$ precedes the integration with respect to $r^{\prime }$ since the former integration will specify the values of the degree, l, and order, m, which will be employed in the latter one. Once again, we stress that these multipole moments are functions of the radius, r, of the ‘shell of observation’. Now we are ready to introduce a relation for the scalar potential of the middle space by taking into account the constituent expressions of both the inside space, relation (7), and the outside space, relation (9). Following all the above argumentation, we introduce the following relation:

(13) $$U_{mid}\left(r\right)=\frac{1}{4\pi \varepsilon_{0}}\sum_{l=0}^{\infty }\frac{4\pi }{2l+1}r^{l}\sum_{m=-l}^{l}q_{l,in}^{m}(r)Y_{l}^{m}(\theta ,\varphi )\;\;\;\;\;\;\;\;\;\;\;\;\;\;\;\;\;\;+\frac{1}{4\pi \varepsilon_{0}}\sum_{l=0}^{\infty }\frac{4\pi }{2l+1}\frac{1}{r^{l+1}}\sum_{m=-l}^{l}q_{l,out}^{m}(r)Y_{l}^{m}(\theta ,\varphi )$$

else

(14) $$U_{mid}\left(r\right)=\frac{1}{4\pi \varepsilon_{0}}\sum_{l=0}^{\infty }\frac{4\pi }{2l+1}\sum_{m=-l}^{l}\left(r^{l}q_{l,in}^{m}\left(r\right)+\frac{q_{l,out}^{m}\left(r\right)}{r^{l+1}}\right)Y_{l}^{m}(\theta ,\varphi )$$

where $q_{l,in}^{m}\left(r\right)$ and $q_{l,out}^{m}\left(r\right)$ are given by relations (11) and (12), respectively.

We argue that relation (14) satisfies all necessary conditions and can serve as a generic, closed-form expression for the reliable calculation of the scalar potential of the middle space in every relevant system.

First, relation (14) satisfies the continuity of the scalar potential at the two spherical interfaces, $r=r_{min}^{\prime }$ and $r=r_{max}^{\prime }$. Indeed, when $r=r_{min}^{\prime },$ the multipole moments $q_{l,out}^{m}(r)$ of relation (12) are zero, while in the calculation of the multipole moments $q_{l,in}^{m}(r)$ with relation (11), the radial integration extends in the entire range, from $r_{min}^{\prime }$ to $r_{max}^{\prime }$. Thus, we easily see that relation (11) retains its original form, relation (8), and relation (14) is identical to relation (7), so that $U_{mid}\left(r\right){|}_{r=r_{min}^{\prime }}=U_{in}\left(r\right){|}_{r=r_{min}^{\prime }}$. Similarly, when $r=r_{max}^{\prime }$ the multipole moments $q_{l,in}^{m}(r)$ of relation (11) are zero, while now in the calculation of the multipole moments $q_{l,out}^{m}(r)$ with relation (12), the radial integration extends in the entire range, from $r_{min}^{\prime }$ to $r_{max}^{\prime }$. Thus, we easily see that now relation (12) retains its original form, relation (10), and relation (14) is identical to relation (9), so that $U_{mid}\left(r\right){|}_{r=r_{max}^{\prime }}=U_{out}\left(r\right){|}_{r=r_{max}^{\prime }}$.

Second, while each of the former constituents of $U_{mid}\left(r\right)$, that is $U_{in}\left(r\right)$ of relations (7) and (8) and $U_{out}\left(r\right)$ of relations (9) and (10), satisfies the Laplace equation, the $U_{mid}\left(r\right)$ defined through relation (14), accompanied by relations (11) and (12), satisfies the Poisson equation.

(15) $$\nabla^{2}U\left(r\right)=-\frac{\rho \left(r\right)}{\varepsilon_{0}}$$

In this context, relation (14) can be brought in the following slightly different version:

(16) $$U_{mid}\left(r\right)=\sum_{l=0}^{\infty }\sum_{m=-l}^{l}\left(\frac{1}{\varepsilon_{0}}\frac{q_{l,in}^{m}\left(r\right)}{2l+1}r^{l}+\frac{1}{\varepsilon_{0}}\frac{q_{l,out}^{m}\left(r\right)}{2l+1}r^{-(l+1)}\right)Y_{l}^{m}(\theta ,\varphi )$$

where $q_{l,in}^{m}\left(r\right)$ and $q_{l,in}^{m}\left(r\right)$ are still given by relations (11) and (12), respectively. Through a comparison of the solution of the Laplace equation, relation (2), with the above relation (16) while recalling relations (11) and (12), we see that we can define the following functions:

(17) $$A_{l,in}^{m}\left(r\right)=\frac{1}{\varepsilon_{0}}\frac{q_{l,in}^{m}\left(r\right)}{2l+1}=\frac{1}{\varepsilon_{0}}\frac{1}{2l+1}\int_{r}^{r_{max}^{\prime }}\frac{1}{{r^{\prime }}^{l-1}}\left(\int_{0}^{\;2\pi }\int_{0}^{\pi }\rho \left(r^{\prime }\right)Y_{l}^{m*}\left(\theta^{\prime },\varphi^{\prime }\right)sin\theta^{\prime }d\theta^{\prime }d\varphi^{\prime }\right)dr^{\prime }$$

and

(18) $$B_{l,out}^{m}\left(r\right)=\frac{1}{\varepsilon_{0}}\frac{q_{l,out}^{m}\left(r\right)}{2l+1}=\frac{1}{\varepsilon_{0}}\frac{1}{2l+1}\int_{r_{min}^{\prime }}^{r}{r^{\prime }}^{l+2}\left(\int_{0}^{\;2\pi }\int_{0}^{\pi }\rho \left(r^{\prime }\right)Y_{l}^{m*}\left(\theta^{\prime },\varphi^{\prime }\right)sin\theta^{\prime }d\theta^{\prime }d\varphi^{\prime }\right)dr^{\prime }$$

In relations (17) and (18), the integration over the angles $\theta^{\prime }$ and $\varphi^{\prime }$ precedes the integration with respect to $r^{\prime }$ since the former integration will specify the values of the degree, l, and order, m, which will be employed in the latter one. Following these definitions, relation (16) can be rewritten in the following form, which is deceptively similar to the solution of the Laplace equation:

(19) $$U_{mid}\left(r\right)=\sum_{l=0}^{\infty }\sum_{m=-l}^{l}\left(A_{l,in}^{m}(r)r^{l}+B_{l,out}^{m}(r)r^{-\left(l+1\right)})Y_{l}^{m}(\theta ,\varphi \right)$$

However, this is not the case; relation (19) satisfies the Poisson equation, relation (15), due to the fact that now the coefficients, $A_{l}^{m}$ and $B_{l}^{m}$, have become functions, $A_{l,in}^{m}(r)$ and $B_{l,out}^{m}(r)$, defined through relations (17) and (18). We prove this fact straightforwardly, below.

## 4. Mathematical Proof of the Expression of the Multipole Expansion of the Scalar Potential in the Middle Space

Starting from the homogeneous Laplace equation (relation (1)), of which the solution is known (relation (2)), we can tackle the nonhomogeneous Poisson equation (relation (15)) and obtain its solution (relation (14) or the equivalent forms, relations (16) and (19)). The proof is based on purely mathematical grounds, by using the method of variation of parameters employed widely in solving ordinary differential equations [39,40]. Briefly, this method is used to find the particular solution of nonhomogeneous differential equations, when the respective solution of the homogeneous case is already known. For a differential equation of second order, the two unknown coefficients of the particular solution can be found through a linear algebraic system of two equations (details can be found in [39,40]). In our case, the homogeneous equation is the Laplace one. The two homogeneous solutions are given in the form of the general terms $A_{l}^{m}\left(r^{l}Y_{l}^{m}\left(\theta ,\varphi \right)\right)$ and $B_{l}^{m}\left(r^{-\left(l+1\right)}Y_{l}^{m}\left(\theta ,\varphi \right)\right)$ (the parentheses highlight the functional part), as shown below:

(20) $$\nabla^{2}U\left(r\right)=\nabla^{2}\sum_{l=0}^{\infty }\sum_{m=-l}^{l}\left(A_{l}^{m}r^{l}+B_{l}^{m}r^{-\left(l+1\right)})Y_{l}^{m}(\theta ,\varphi \right)\;\;\;\;\;\;=\sum_{l=0}^{\infty }\sum_{m=-l}^{l}\left(A_{l}^{m}\nabla^{2}\left(r^{l}Y_{l}^{m}\left(\theta ,\varphi \right)\right)\;\;\;\;\;\;\;+B_{l}^{m}\nabla^{2}\left(r^{-\left(l+1\right)}Y_{l}^{m}\left(\theta ,\varphi \right)\right)\right)=0$$

Based on the method of variation of parameters, the constant coefficients, $A_{l}^{m}$ and $B_{l}^{m}$, should become functions of the position vector, $A_{l,in}^{m}(r)$ and $B_{l,out}^{m}(r)$. Once we determine these coefficient functions, the expression of the scalar potential found above for the middle space, $U_{mid}\left(r\right)$, should satisfy the Poisson equation as follows:

(21) $$\nabla^{2}U_{mid}\left(r\right)=\nabla^{2}\sum_{l=0}^{\infty }\sum_{m=-l}^{l}\left(A_{l,in}^{m}(r)r^{l}+B_{l,out}^{m}(r)r^{-\left(l+1\right)})Y_{l}^{m}(\theta ,\varphi \right)\;\;\;=\sum_{l=0}^{\infty }\sum_{m=-l}^{l}\left(\nabla^{2}\left(A_{l,in}^{m}\left(r\right)r^{l}Y_{l}^{m}\left(\theta ,\varphi \right)\right)\;\;\;\;\;\;+\nabla^{2}\left(B_{l,out}^{m}\left(r\right)r^{-\left(l+1\right)}Y_{l}^{m}\left(\theta ,\varphi \right)\right)\right)=-\frac{\rho \left(r\right)}{\varepsilon_{0}}$$

The two unknown coefficient functions, $A_{l,in}^{m}(r)$ and $B_{l,out}^{m}(r)$, should be determined through the linear algebraic system by means of the following two equations:

(22) $$\left(\nabla A_{l,in}^{m}\left(r\right)\right)\left(r^{l}Y_{l}^{m}\left(\theta ,\varphi \right)\right)+\left(\nabla B_{l,out}^{m}\left(r\right)\right)\left(r^{-\left(l+1\right)}Y_{l}^{m}\left(\theta ,\varphi \right)\right)=0$$

and

(23) $$\left(\nabla A_{l,in}^{m}\left(r\right)\right)\cdot \left(\nabla \left(r^{l}Y_{l}^{m}\left(\theta ,\varphi \right)\right)\right)+\left(\nabla B_{l,out}^{m}\left(r\right)\right)\cdot \left(\nabla \left(r^{-\left(l+1\right)}Y_{l}^{m}\left(\theta ,\varphi \right)\right)\right)=-\frac{\rho \left(r\right)}{\varepsilon_{0}}$$

From Equation (22), we obtain the following:

(24) $$\left(\nabla A_{l,in}^{m}\left(r\right)\right)=-r^{-\left(2l+1\right)}\left(\nabla B_{l,out}^{m}\left(r\right)\right)$$

By using the above relation, relation (23) becomes the following:

(25) $$\left(\nabla B_{l,out}^{m}\left(r\right)\right)\cdot \left(\nabla \left(r^{l}Y_{l}^{m}\left(\theta ,\varphi \right)\right)\right)\left(-r^{-\left(2l+1\right)}\right)+\left(\nabla B_{l,out}^{m}\left(r\right)\right)\cdot \left(\nabla \left(r^{-\left(l+1\right)}Y_{l}^{m}\left(\theta ,\varphi \right)\right)\right)=-\frac{\rho \left(r\right)}{\varepsilon_{0}}$$

The coefficient functions, $B_{l,out}^{m}\left(r\right)$, can be found from this relation. To this end, $\nabla \left(r^{l}Y_{l}^{m}\left(\theta ,\varphi \right)\right)$ and $\nabla \left(r^{-\left(l+1\right)}Y_{l}^{m}\left(\theta ,\varphi \right)\right)$ should be determined. To facilitate the algebraic calculations, we write the ∇ operator in the following form:

(26) $$\nabla =\hat{r}\frac{\partial }{\partial r}+\hat{\theta }\frac{1}{r}\frac{\partial }{\partial \theta }+\hat{\varphi }\frac{1}{rsin\theta }\frac{\partial }{\partial \varphi }=\hat{r}\frac{\partial }{\partial r}+\frac{1}{r}\left(\hat{\theta }\frac{\partial }{\partial \theta }+\hat{\varphi }\frac{1}{sin\theta }\frac{\partial }{\partial \varphi }\right)=\nabla_{r}+\frac{1}{r}\nabla_{\theta \varphi }$$

Thus, $\nabla \left(r^{l}Y_{l}^{m}\left(\theta ,\varphi \right)\right)$ easily becomes the following:

(27) $$\nabla \left(r^{l}Y_{l}^{m}\left(\theta ,\varphi \right)\right)=\hat{r}lr^{l-1}Y_{l}^{m}\left(\theta ,\varphi \right)+r^{l-1}\left(\nabla_{\theta \varphi }Y_{l}^{m}\left(\theta ,\varphi \right)\right)$$

Similarly, $\nabla \left(r^{-\left(l+1\right)}Y_{l}^{m}\left(\theta ,\varphi \right)\right)$ has the following form:

(28) $$\nabla \left(r^{-\left(l+1\right)}Y_{l}^{m}\left(\theta ,\varphi \right)\right)=-\hat{r}(l+1)r^{-(l+2)}Y_{l}^{m}\left(\theta ,\varphi \right)+r^{-(l+2)}\left(\nabla_{\theta \varphi }Y_{l}^{m}\left(\theta ,\varphi \right)\right)$$

Inserting relations (27) and (28) into relation (25), after simple algebra, we obtain the following:

(29) $$\frac{\partial B_{l,out}^{m}\left(r\right)}{\partial r}Y_{l}^{m}\left(\theta ,\varphi \right)=\frac{1}{2l+1}r^{l+2}\frac{\rho \left(r\right)}{\varepsilon_{0}}$$

Substituting the above relation into relation (24), we obtain the following:

(30) $$\frac{\partial A_{l,in}^{m}\left(r\right)}{\partial r}Y_{l}^{m}\left(\theta ,\varphi \right)=-\frac{1}{2l+1}r^{-(l-1)}\frac{\rho \left(r\right)}{\varepsilon_{0}}$$

At this point, we make the one and only assumption in our argumentation: we argue that the coefficient functions depend only on the radial coordinate, thus, $A_{l,in}^{m}\left(r\right)\to A_{l,in}^{m}\left(r\right)$ and $B_{l,out}^{m}\left(r\right)\to B_{l,out}^{m}\left(r\right)$. This assumption is documented by the following argument: let us consider the case where, except for r, the coefficients $A_{l,in}^{m}$ and $B_{l,out}^{m}$ depend on the polar and azimuthal angles as well, i.e., $A_{l,in}^{m}\left(r\right)=A_{l,in}^{m}\left(r,\theta ,\varphi \right)$ and $B_{l,out}^{m}\left(r\right)=B_{l,out}^{m}(r,\theta ,\varphi )$. Then, the solution should have the following form: $U_{mid}\left(r\right)=\sum_{l=0}^{\infty }\sum_{m=-l}^{l}\left(A_{l,in}^{m}\left(r,\theta ,\varphi \right)r^{l}+B_{l,out}^{m}\left(r,\theta ,\varphi \right)r^{-\left(l+1\right)}\right)Y_{l}^{m}(\theta ,\varphi )$. This relation has the obvious disadvantage that it overexpresses the dependence on the polar, θ, and azimuthal, φ, angles in all terms, both inside and outside the parentheses. Thus, this relation practically violates the separation-of-variables form that the desired solution should obey. On the other hand, when the coefficients $A_{l,in}^{m}$ and $B_{l,out}^{m}$ depend only on r, the obtained solution preserves the separation-of-variables form, as it should (see below).

Accordingly, using relations (29) and (30), by multiplying with $Y_{l}^{m*}\left(\theta ,\varphi \right)$, appropriate integration, and by using the normalization relation (4), we obtain the following:

(31) $$A_{l,in}^{m}\left(r\right)=\frac{1}{\varepsilon_{0}}\frac{1}{2l+1}\int_{r}^{r_{max}^{\prime }}\int_{0}^{\;2\pi }\int_{0}^{\pi }\rho \left(r\right)r^{-(l+1)}Y_{l}^{m*}\left(\theta ,\varphi \right)r^{2}sin\theta drd\theta d\varphi$$

and

(32) $$B_{l,out}^{m}\left(r\right)=\frac{1}{\varepsilon_{0}}\frac{1}{2l+1}\int_{r_{min}^{\prime }}^{r}\int_{0}^{\;2\pi }\int_{0}^{\pi }\rho \left(r\right)r^{l}Y_{l}^{m*}\left(\theta ,\varphi \right)r^{2}sin\theta drd\theta d\varphi$$

These relations can be rewritten in the final form:

(33) $$A_{l,in}^{m}\left(r\right)=\frac{1}{\varepsilon_{0}}\frac{1}{2l+1}\int_{r}^{r_{max}^{\prime }}{r^{\prime }}^{-(l-1)}\left(\int_{0}^{\;2\pi }\int_{0}^{\pi }\rho \left(r^{\prime }\right)Y_{l}^{m*}\left(\theta^{\prime },\varphi^{\prime }\right)sin\theta^{\prime }d\theta^{\prime }d\varphi^{\prime }\right)dr^{\prime }$$

and

(34) $$B_{l,out}^{m}\left(r\right)=\frac{1}{\varepsilon_{0}}\frac{1}{2l+1}\int_{r_{min}^{\prime }}^{r}{r^{\prime }}^{l+2}\left(\int_{0}^{\;2\pi }\int_{0}^{\pi }\rho \left(r^{\prime }\right)Y_{l}^{m*}\left(\theta^{\prime },\varphi^{\prime }\right)sin\theta^{\prime }d\theta^{\prime }d\varphi^{\prime }\right)dr^{\prime }$$

With respect to relations (31) and (32), in the above relations (33) and (34), we rearranged the triple integration and introduced the primed variables $r^{\prime }=(r^{\prime },\theta^{\prime },\varphi^{\prime })$ in the integrals (referring to the coordinates of the source) to avoid any misunderstanding of non-experts in the field. Regarding the radial integration, since the $A_{l,in}^{m}\left(r\right)$ coefficient function refers to the inside space, the maximum integration interval should range from r to ∞. Similarly, since the $B_{l,out}^{m}\left(r\right)$ coefficient function refers to the outside space, the maximum integration interval should range from 0 to r. However, in our case, by recalling Figure 1 and Figure 2, we realize that the radial integration should be limited from r to $r_{max}^{\prime }$ in relations (31) and (33) and from $r_{min}^{\prime }$ to r in relations (32) and (34), since outside these intervals, the volume charge density is zero, $\rho \left(r\right)=0$. The same holds for the integration over the angles (actually, the integration should be confined inside the volume of the charge density, see Figure 1 and Figure 2). By making a comparison of these relations with relations (17) and (18), we conclude that indeed relation (19) satisfies the Poisson equation, relation (15).

Notice that in the above procedure, we have completely bypassed the standard method in which the multipole expansion of ${\left|r-r^{\prime }\right|}^{-1}$ is used in the generalized law of Coulomb [36,37,38]. Instead, here, we proved, purely mathematically, the true character of the multipole expansion of the scalar potential in the middle space, $U_{mid}\left(r\right)$; it is actually the solution of the nonhomogeneous Poisson equation (that is, in spaces wherein volume charge densities, $\rho \left(r\right)$, reside). This is a standalone, important finding of our work.

## 5. Case of Azimuthal Symmetry

The above analysis described the most general case where the system depends on all (r,θ,φ). The case of azimuthal symmetry is relatively easier to handle. With simple algebra, we see that in such cases, the above relations (19), (33), and (34) of the multipole expansion in the middle space attain the following form:

(35) $$U_{mid}\left(r\right)=\sum_{l=0}^{\infty }\left(A_{l,in}(r)r^{l}+B_{l,out}(r)r^{-\left(l+1\right)})P_{l}(cos\theta \right)$$

where

(36) $$A_{l,in}\left(r\right)=\frac{1}{2\varepsilon_{0}}\int_{r}^{r_{max}^{\prime }}{r^{\prime }}^{-(l-1)}\left(\int_{0}^{\pi }\rho \left(r^{\prime }\right)P_{l}(cos\theta^{\prime })sin\theta^{\prime }d\theta^{\prime }\right)dr^{\prime }$$

and

(37) $$B_{l,out}\left(r\right)=\frac{1}{2\varepsilon_{0}}\int_{r_{min}^{\prime }}^{r}{r^{\prime }}^{l+2}\left(\int_{0}^{\pi }\rho \left(r^{\prime }\right)P_{l}(cos\theta^{\prime })sin\theta^{\prime }d\theta^{\prime }\right)dr^{\prime }$$

In the above expressions, $P_{l}$ refers to the Legendre polynomials. In relations (36) and (37), the integration over the angle $\theta^{\prime }$ precedes the integration with respect to $r^{\prime }$ since the former one specifies the values of the degree, l, which will be employed in the latter.

## 6. Our Method as an Effective Solver of the Poisson Equation in Dielectric and Magnetic Materials, Both Homogeneous and Nonhomogeneous

Until now, we referred to the scalar potential for the case of sources in electricity (e.g., a volume charge density). Here, we clarify that our argumentation embraces both conducting (i.e., free charges, $\rho_{f}\left(r\right)$) and dielectric (i.e., bound charges, $\rho_{b}\left(r\right)$) materials, so that the (total) volume charge density, $\rho \left(r\right)=\rho_{f}\left(r\right)+\rho_{b}\left(r\right)$, should be used in all the above mathematical analysis. In the category of dielectrics, both non-permanent and permanent types are included since, in all instances, a bound volume charge density is defined by the electric polarization, P(r), through the following general relation [37,38,41,42]:

(38) $$\rho_{b}\left(r\right)=-\nabla \cdot P(r)$$

Similarly, the above analysis adapts to magnetic materials. In this case, a volume density of the so-called magnetic pseudocharges is defined by the magnetic polarization, M(r), through the following general relation [37,38,41,42]:

(39) $$\rho_{b,m}\left(r\right)=-\nabla \cdot M(r)$$

where the lower index ‘m’ addresses magnetism. Once in magnetism, free charges do not exist, $\rho_{f,m}\left(r\right)=0$, and the bound pseudocharges, $\rho_{b,m}\left(r\right)$, defined above, are the only sources to be considered. Below, we provide the final expressions obtained by the above mathematical analysis, for the case of magnetic materials, when the system depends on all (r,θ,φ):

(40) $$U_{mid,m}\left(r\right)=\sum_{l=0}^{\infty }\sum_{m=-l}^{l}\left(A_{l,in,m}^{m}(r)r^{l}+B_{l,out,m}^{m}(r)r^{-\left(l+1\right)})Y_{l}^{m}(\theta ,\varphi \right)$$

where

(41) $$A_{l,in,m}^{m}\left(r\right)=\frac{1}{2l+1}\int_{r}^{r_{max}^{\prime }}\frac{1}{{r^{\prime }}^{l-1}}\left(\int_{0}^{\;2\pi }\int_{0}^{\pi }\rho_{b,m}\left(r^{\prime }\right)Y_{l}^{m*}\left(\theta^{\prime },\varphi^{\prime }\right)sin\theta^{\prime }d\theta^{\prime }d\varphi^{\prime }\right)dr^{\prime }$$

and

(42) $$B_{l,out,m}^{m}\left(r\right)=\frac{1}{2l+1}\int_{r_{min}^{\prime }}^{r}{r^{\prime }}^{l+2}\left(\int_{0}^{\;2\pi }\int_{0}^{\pi }\rho_{b,m}\left(r^{\prime }\right)Y_{l}^{m*}\left(\theta^{\prime },\varphi^{\prime }\right)sin\theta^{\prime }d\theta^{\prime }d\varphi^{\prime }\right)dr^{\prime }$$

As was discussed for the case of electricity, in relations (40) and (41), which hold for magnetism, the integration over the angles $\theta^{\prime }$ and $\varphi^{\prime }$ precedes the integration with respect to $r^{\prime }$ since the former integration will specify the values of the degree, l, and order, m, which will be employed in the latter one.

The respective case of azimuthal symmetry is described by the following relations:

(43) $$U_{mid,m}\left(r\right)=\sum_{l=0}^{\infty }\left(A_{l,in,m}^{m}(r)r^{l}+B_{l,out,m}^{m}(r)r^{-\left(l+1\right)})P_{l}(cos\theta \right)$$

where

(44) $$A_{l,in,m}\left(r\right)=\frac{1}{2}\int_{r}^{r_{max}^{\prime }}{r^{\prime }}^{-(l-1)}\left(\int_{0}^{\pi }\rho_{b,m}\left(r^{\prime }\right)P_{l}(cos\theta^{\prime })sin\theta^{\prime }d\theta^{\prime }\right)dr^{\prime }$$

and

(45) $$B_{l,out,m}\left(r\right)=\frac{1}{2}\int_{r_{min}^{\prime }}^{r}{r^{\prime }}^{l+2}\left(\int_{0}^{\pi }\rho_{b,m}\left(r^{\prime }\right)P_{l}(cos\theta^{\prime })sin\theta^{\prime }d\theta^{\prime }\right)dr^{\prime }$$

In relations (44) and (45), the integration over the angle $\theta^{\prime }$ precedes the integration with respect to $r^{\prime }$ since the former one specifies the values of the degree, l, which will be employed in the latter.

Finally, we should clarify that our mathematical description is applicable to both homogeneous and nonhomogeneous sources. Actually, this is a nice, inherent advantage of our method; it can be employed to solve the Poisson equation rather easily, even when the source is nonhomogeneous, whether it is of free or bound origin. This is documented in representative cases of dielectric and magnetic materials, drawn from the literature, as discussed below.

## 7. The Multipole Expansion as an Effective Solver of the Poisson Equation and Its Applicability in Science and Engineering

Above, we theoretically documented that the multipole expansion of the scalar potential in the middle space (defined by relations (17)–(19) for the general case and by relations (35)–(37) for the case of azimuthal symmetry) satisfies the Poisson equation. We argue that this has immediate implications for calculations needed in applications. Below, we test our theoretical notion against standard systems found in applications in the literature and prove that, indeed, the expression of $U_{mid}\left(r\right)$ introduced here is a generic and effective solver of problems referring to spaces wherein a volume density of charge, $\rho \left(r\right)$, resides. We note that in the following examples, we use the notation $U_{in}\left(r\right)$ for the scalar potential (though in the parent relations (19) and (35), we use the notation $U_{mid}\left(r\right)$) to highlight the fact that we calculate the electric potential inside the charge density. Specifically, below, we utilize in detail our generic expressions in some representative examples drawn from three wide thematic categories of physics, namely atomic, solid state, and materials science. We document that the expressions of the multipole expansion of the scalar potential for the middle space introduced in this work can be employed straightforwardly to solve the Poisson equation, quite effectively with relatively little effort, thus obtaining the scalar potential in spaces wherein volume charges reside. Though cases based on electric charges (both free and bound) are mostly reviewed, examples wherein magnetic pseudocharges (bound) are involved are also considered.

### 7.1. Scalar Potential Obtained from the Charge Density of Atomic Orbitals

In this case, we can obtain the electric scalar potential, U(r), directly from the charge density, $\rho_{nlm}\left(r\right)\sim {\left|\Psi_{nlm}\left(r\right)\right|}^{2}$, of the electrons participating in an atomic orbital of quantum numbers (n,l,m) (where $\Psi_{nlm}\left(r\right)$ is the respective electron wave function). We will investigate this issue for two representative examples of the hydrogen atom for which the exact information is available [37,43,44,45,46].

#### 7.1.1. Electronic Ground State $\left(n,l,m\right)=(1,0,0)$ in the Hydrogen Atom

**Figure 3:**
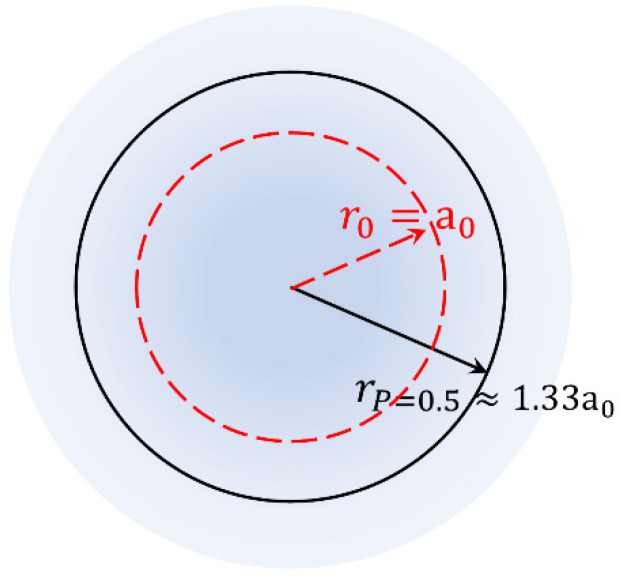
Illustrated scheme of the wave function $\Psi_{100}\left(r\right)$ (the color code refers to the magnitude of $\Psi_{100}\left(r\right)$; see relation (46)). The characteristic radius $r_{P=0.5}\approx 1.33a_{0}$ (black, solid circle) is defined through satisfying the equation $\int_{V}\left|\Psi_{100}\left(r\right)\right|dV=P$ for the prescribed value of probability, P=1/2. The characteristic radius $r_{0}=a_{0}$ (red, dashed circle) is defined through the maximization of the radial probability density function, ${\left|\Psi_{100}\left(r\right)\right|}^{2}r^{2}=(1/\pi )(1/a_{0}^{3})r^{2}e^{-2r/a_{0}}$.

In the first primitive case, the charge density exhibits only radial dependence. This is the 1s state of the electron in the hydrogen atom, which is characterized by the wave function given by the following relation:

(46) $$\Psi_{100}\left(r\right)=\frac{1}{\sqrt{\pi }}\frac{1}{a_{0}^{3/2}}e^{-r/a_{0}}$$

and the respective charge density, $\rho_{1s}\left(r\right)=q_{e}{\left|\Psi_{100}\left(r\right)\right|}^{2}$, given by the following relation:

(47) $$\rho_{1s}\left(r\right)=\frac{q_{e}}{\pi a_{0}^{3}}e^{-2r/a_{0}}$$

where $a_{0}=4\pi \varepsilon_{0}\hbar^{2}/q_{e}^{2}m_{e}$ is the Bohr radius ($q_{e}$ and $m_{e}$ refer to the charge and mass of the electron). In Figure 3 below, we show an illustration of the ground state wave function, $\Psi_{100}\left(r\right)$. Following relation (46), the scheme illustrates the case where the wave function exhibits only an exponential dependence on r. Two characteristic radii are shown. The first one, $r_{P}$, is specified by the demand that the electron should be observed within the volume enclosed by $r_{P}$ at a prescribed probability P. In mathematical terms $\int_{V}\left|\Psi_{100}\left(r\right)\right|dV=P,$ which for P=1/2 leads to the equation $(2r^{2}+2a_{0}r+a_{0}^{2})e^{-2r/a_{0}}=a_{0}^{2}/2$. The numerical solution of this relation results in $r_{P=0.5}\approx 1.33a_{0}$. The second one, $r_{0}$, is termed the most probable radius of electron orbiting and is defined as the maximum observed in the radial probability density function ${\left|\Psi_{100}\left(r\right)\right|}^{2}r^{2}=(1/\pi )(1/a_{0}^{3})r^{2}e^{-2r/a_{0}}$, which results in $r_{0}=a_{0}$ (see below and [45,46]).

In this particular case, the charge density, $\rho_{1s}\left(r\right)$, has both polar and azimuthal symmetry, depending solely on r, thus being $\rho_{1s}\left(r\right)$. We use relations (35)–(37), where apparently only the term l=0 should be inserted to calculate the respective electric scalar potential (obviously, $A_{l,in}\left(r\right)=B_{l,out}\left(r\right)=0$ for l≠0). First, from relations (36) and (37), we easily obtain the coefficient functions

(48) $$A_{0,in}\left(r\right)=\frac{q_{e}}{4\pi \varepsilon_{0}a_{0}^{2}}(2r+a_{0})e^{-2r/a_{0}}$$

and

(49) $$B_{0,out}\left(r\right)=\frac{q_{e}}{4\pi \varepsilon_{0}a_{0}^{2}}\left(a_{0}^{2}-\left(2r^{2}+2a_{0}r+a_{0}^{2}\right)e^{-2r/a_{0}}\right)$$

**Figure 4:**
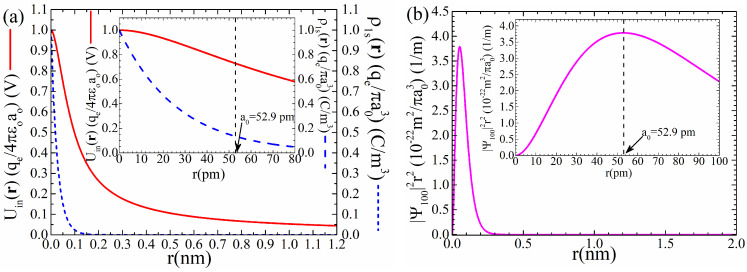
(**a**) Scalar potential, $U_{in}\left(r\right)$, relation (50), together with the respective charge density, $\rho_{1s}\left(r\right)$, relation (47). (**b**) Radial probability density function, ${\left|\Psi_{100}\left(r\right)\right|}^{2}r^{2}=(1/\pi )(1/a_{0}^{3})r^{2}e^{-2r/a_{0}}$. In both cases, the main panel shows the graph in an extended range, while the inset focuses on a small range close to zero. In panel (**a**), the electron charge, $q_{e}$, should be accompanied by the minus sign. In both panels (**a**,**b**), the value $a_{0}=52.9\;pm$ was used for the Bohr radius.

Then, substituting the above $A_{0,in}\left(r\right)$ and $B_{0,out}\left(r\right)$ into relation (35), we obtain the electric scalar potential produced by the electron of the 1s state in the hydrogen atom:

(50) $$U_{in}\left(r\right)=\frac{q_{e}}{4\pi \varepsilon_{0}a_{0}}\left(\frac{a_{0}}{r}-\left(1+\frac{a_{0}}{r}\right)e^{-2r/a_{0}}\right)$$

where $q_{e}$ should be accompanied by the minus sign. In Figure 4, panel (a), we present a detailed plot of $U_{in}\left(r\right)$, relation (50), together with the respective charge density, $\rho_{1s}\left(r\right)$, relation (47). We see that both $U_{in}\left(r\right)$ and $\rho_{1s}\left(r\right)$ exhibit a conventional behavior of a monotonously decreasing function, without any extrema. Thus, $U_{in}\left(r\right)$ follows the behavior of $\rho_{1s}\left(r\right),$ which resembles an electron effectively located mainly at the origin of the coordinate system, r=0, that is, at the nucleus. In contrast, from a quantum mechanical point of view, we expect the behavior shown by the radial probability density function, ${\left|\Psi_{100}\left(r\right)\right|}^{2}r^{2}$, observed in panel (b). Now, the most probable radius of the electron orbiting is observed at the maximum located at $r=a_{0}$, as it should be [45,46].

The scalar potential, $U_{in}\left(r\right)$, relation (50), obtained by the methodology introduced here, is exact and reliable. For instance, in [37] (see page 50, problem 1.5), the scalar potential was obtained for the entire neutral hydrogen atom in its ground state (that is, taking into account the contribution of both the proton of the nucleus and the electron orbiting in the 1s state). Here, we isolated effectively the scalar potential produced solely by the 1s electron. A comparison between the two results, the one obtained in [37] and the one obtained here, clarifies their consistency.

#### 7.1.2. Electronic State $\left(n,l,m\right)=(2,1,\pm 1)$ in the Hydrogen Atom

We proceed with a case where the charge density also depends on the polar angle, θ. Such a case is the 2p state of the electron in the hydrogen atom, expressed by the following wave function:

(51) $$\Psi_{21\pm 1}\left(r\right)=\mp \frac{1}{8\sqrt{\pi }}\frac{1}{a_{0}^{3/2}}\frac{r}{a_{0}}e^{-r/2a_{0}}sin\theta e^{\pm i\varphi }$$

while the respective charge density, $\rho_{2p}\left(r\right)=q_{e}{\left|\Psi_{21\pm 1}\left(r\right)\right|}^{2}$, is given by the following relation:

(52) $$\rho_{2p}\left(r\right)=\frac{q_{e}}{64\pi a_{0}^{3}}\frac{r^{2}}{a_{0}^{2}}e^{-r/a_{0}}{sin}^{2}\theta$$

**Figure 5:**
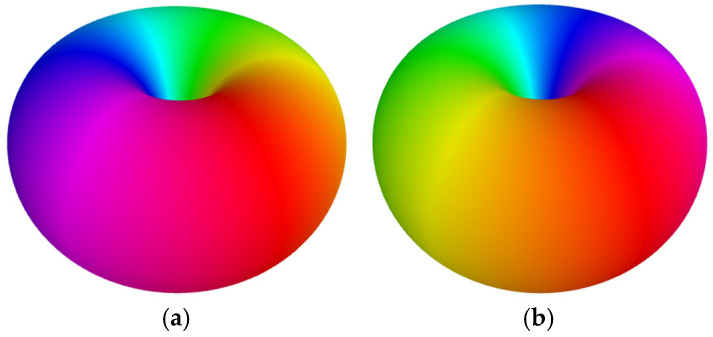
Illustrated schemes of the wave function (**a**) $\Psi_{211}\left(r\right)$ and (**b**) $\Psi_{21-1}\left(r\right)$ (the color code refers to the phase of $\Psi_{21\pm 1}\left(r\right)$; see relation (48)). The electron is found within the volume enclosed by the isodensity surface $\left|\Psi_{21\pm 1}\left(r\right)\right|$, for a prescribed value of probability, P (in the present cases P=0.5) [47].

In Figure 5 below, we show an illustration of the 2p state, $\Psi_{21\pm 1}\left(r\right)$ [47]. In this case, $\left|\Psi_{21\pm 1}\left(r\right)\right|$ exhibits both radial and polar dependence, as evidenced by relation (48).

In this more interesting case, the charge density, $\rho_{2p}\left(r\right)$, has only azimuthal symmetry, depending on both r and θ, thus being $\rho_{2p}\left(r,\theta \right)$. We use relations (35)–(37) to calculate the electric scalar potential. To this end, first, we expand $\rho_{2p}\left(r\right)$ on the basis of Legendre so that we obtain the following:

(53) $$\rho_{2p}\left(r\right)=\frac{q_{e}}{64\pi a_{0}^{3}}\frac{r^{2}}{a_{0}^{2}}e^{-r/a_{0}}{sin}^{2}\theta =\frac{q_{e}}{64\pi a_{0}^{3}}\frac{r^{2}}{a_{0}^{2}}e^{-r/a_{0}}\left(\frac{2}{3}\;P_{0}\left(cos\theta \right)-\frac{2}{3}\;P_{2}\left(cos\theta \right)\right)$$

where $P_{0}\left(cos\theta \right)=1$ and $P_{2}\left(cos\theta \right)=1/2\left(3{cos}^{2}\theta -1\right)$ [37,46]. We see that now we will have terms referring to l=0 and l=2, that is, $A_{0,in}\left(r\right)$, $A_{2,in}\left(r\right)$, $B_{0,out}\left(r\right),$ and $B_{2,out}\left(r\right)$ (obviously, $A_{l,in}\left(r\right)=B_{l,out}\left(r\right)=0$ for l≠0,2). Inserting relation (53) into relations (36) and (37), we obtain the following coefficient functions:

(54) $$A_{0,in}\left(r\right)=\frac{1}{24}\frac{q_{e}}{4\pi \varepsilon_{0}}\frac{1}{a_{0}^{4}}(r^{3}+3a_{0}r^{2}+6a_{0}^{2}r+6a_{0}^{3})e^{-r/a_{0}}$$

for l=0,

(55) $$A_{2,in}\left(r\right)=-\frac{1}{120}\frac{q_{e}}{4\pi \varepsilon_{0}}\frac{1}{a_{0}^{4}}(r+a_{0})e^{-r/a_{0}}$$

for l=2, while

(56) $$B_{0,out}\left(r\right)=\frac{1}{24}\frac{q_{e}}{4\pi \varepsilon_{0}}\frac{1}{a_{0}^{5}}\left[24a_{0}^{5}-\left(a_{0}r^{4}+4a_{0}^{2}r^{3}+12a_{0}^{3}r^{2\;}+24a_{0}^{4}r+24a_{0}^{5}\right)e^{-r/a_{0}}\right]$$

for l=0, and

(57) $$B_{2,out}\left(r\right)=-\frac{2}{15}\frac{q_{e}}{4\pi \varepsilon_{0}}\frac{1}{a_{0}^{5}}\left[720a_{0}^{7}-\left(a_{0}r^{6}+6a_{0}^{2}r^{5}+30a_{0}^{3}r^{4}+120a_{0}^{4}r^{3}+360a_{0}^{5}r^{2}+720a_{0}^{6}r+720a_{0}^{7}\right)e^{-r/a_{0}}\right]$$

for the last coefficient with l=2.

Substituting the above coefficient functions, $A_{0,in}\left(r\right)$, $A_{2,in}\left(r\right)$, $B_{0,out}\left(r\right),$ and $B_{2,out}\left(r\right)$ into relation (35), we obtain the electric scalar potential produced by the electron of the 2p state in the hydrogen atom:

(58) $$U_{in}\left(r\right)=\frac{q_{e}}{4\pi \varepsilon_{0}}\left[\frac{1}{r}-\frac{1}{24}\left(\frac{r^{2}}{a_{0}^{3}}+6\frac{r}{a_{0}^{2}}+18\frac{1}{a_{0}}+24\frac{1}{r}\right)e^{-r/a_{0}}\right]P_{0}\left(cos\theta \right)\;+\frac{q_{e}}{4\pi \varepsilon_{0}}\left[-6\frac{a_{0}^{2}}{r^{3}}+\frac{5}{120}\left(\frac{r^{2}}{a_{0}^{3}}+6\frac{r}{a_{0}^{2}}+24\frac{1}{a_{0}}+72\frac{1}{r}+144\frac{a_{0}}{r^{2}}+144\frac{a_{0}^{2}}{r^{3}}\right)e^{-r/a_{0}}\right]P_{2}(cos\theta )$$

else

(59) $$U_{in}\left(r\right)=\frac{q_{e}}{4\pi \varepsilon_{0}}\left[\frac{1}{r}-\frac{1}{24}\left(\frac{r^{2}}{a_{0}^{3}}+6\frac{r}{a_{0}^{2}}+18\frac{1}{a_{0}}+24\frac{1}{r}\right)e^{-r/a_{0}}\right]\;+\frac{q_{e}}{4\pi \varepsilon_{0}}\left[-6\frac{a_{0}^{2}}{r^{3}}+\frac{5}{120}\left(\frac{r^{2}}{a_{0}^{3}}+6\frac{r}{a_{0}^{2}}+24\frac{1}{a_{0}}+72\frac{1}{r}+144\frac{a_{0}}{r^{2}}+144\frac{a_{0}^{2}}{r^{3}}\right)e^{-r/a_{0}}\right]\frac{1}{2}\left(3{cos}^{2}\theta -1\right)$$

We note that in the above relation, $q_{e}$ should be accompanied by the minus sign.

**Figure 6:**
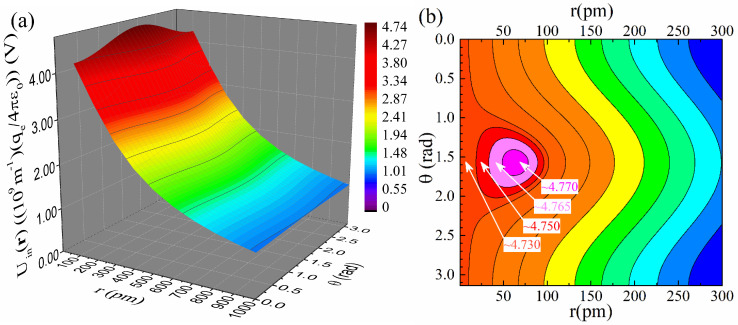
Detailed plots of the scalar potential, $U_{in}\left(r\right)$, relation (59), in (**a**) 3D form for an extended range and (**b**) contour form for a relatively smaller range. The color code of the contour plot follows that of the 3D one, except for the regime around the weak maximum located at $\left(r,\theta \right)\approx (65\;\mathrm{pm},1.57\;\mathrm{rad})$, on the order of 0.96%, where a different color code has been used locally to highlight its existence. The value $a_{0}=52.9\;\mathrm{pm}$ was used for the Bohr radius.

Figure 6a,b present plots of $U_{in}\left(r\right)$, relation (59), in 3D and contour form, respectively. The 3D plot, panel (a), renders an extended range, while the contour one, panel (b), focuses on a smaller range. At first sight, the scalar potential exhibits a somewhat conventional behavior, being maximum at the origin, that is, at the nucleus, while it decreases monotonously with radius, r, as evidenced in panel (a). However, a closer inspection of the data simulated with high resolution reveals that $U_{in}\left(r\right)$ exhibits a maximum located off-nucleus at $\left(r,\theta \right)\approx (65\;\mathrm{pm},1.57\;\mathrm{rad})$. As evidenced by the displayed values of $U_{in}\left(r\right)$ in the vicinity of $\left(65\;\mathrm{pm},1.57\;\mathrm{rad}\right),$ the maximum is very weak, thus difficult to discern without simulations of high resolution and careful assessment of the respective data. On a quantitative basis, the percentage variation of $U_{in}\left(r\right)$ in the vicinity of the maximum is $((U_{in}\left(65\;\mathrm{pm},1.57\;\mathrm{rad}\right)-U_{in}\left(0\;\mathrm{pm},1.57\;\mathrm{rad}\right))/U_{in}\left(65\;\mathrm{pm},1.57\;\mathrm{rad}\right))\times 100\%=\left((4.7717-4.7259)/4.7717\right)\times 100\%=0.96\%$.

**Figure 7:**
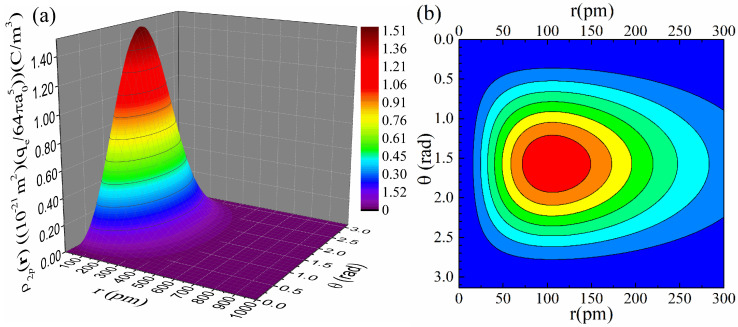
Detailed plots of the charge density, $\rho_{2p}\left(r\right)$, relation (52), in (**a**) 3D form for an extended range and (**b**) contour form for a relatively smaller range. The color code of the contour plot follows that of the 3D one. The value $a_{0}=52.9\;\mathrm{pm}$ was used for the Bohr radius.

Figure 7a,b present the relevant plots of the charge density, $\rho_{2p}\left(r\right)$, relation (52), in 3D and contour form, respectively. From both the 3D plot of panel (a), which renders an extended range, and the contour one of panel (b), which focuses on a smaller range, we see that $\rho_{2p}\left(r\right)$ exhibits a pronounced non-monotonous behavior with a maximum located away from the nucleus at $\left(r,\theta \right)\approx (101\;\mathrm{pm},1.57\;\mathrm{rad})$.

**Figure 8:**
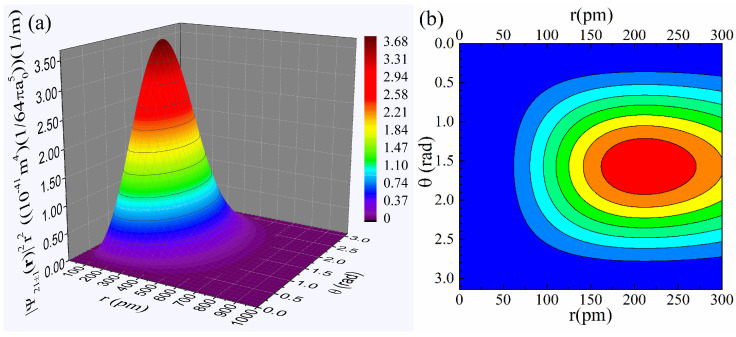
Detailed plots of the probability density function, ${\left|\Psi_{21\pm 1}\left(r\right)\right|}^{2}r^{2}$ in (**a**) 3D form for an extended range and (**b**) contour form for a relatively smaller range. The color code of the contour plot follows that of the 3D one. The value $a_{0}=52.9\;\mathrm{pm}$ was used for the Bohr radius.

Finally, Figure 8a,b present the plots of the probability density function, ${\left|\Psi_{21\pm 1}\left(r\right)\right|}^{2}r^{2}=(1/64\pi a_{0}^{5})r^{4}e^{-r/a_{0}}{sin}^{2}\theta$, in 3D and contour form as well. From both the 3D plot of panel (a), which renders an extended range, and the contour one of panel (b), which focuses on a smaller range, we see that the probability density function exhibits practically the same pronounced non-monotonous behavior of $\rho_{2p}\left(r\right)$, with a maximum located even farther from the nucleus at $\left(r,\theta \right)\approx (212\;\mathrm{pm},1.57\;\mathrm{rad})$.

These plots can be compared directly since in all panels (a) and all panels (b), the same scale was used for both the radial and the polar coordinates (0 pm≤r≤1000 pm in all panels (a) and 0 pm≤r≤300 pm in all panels (b), while 0 rad≤θ≤3.14 rad in all cases). Thus, from these plots becomes apparent that the charge density, $\rho_{2p}\left(r\right)={q_{e}|\Psi_{21\pm 1}\left(r\right)|}^{2}$ and the probability density function, ${\left|\Psi_{21\pm 1}\left(r\right)\right|}^{2}r^{2}$, exhibit the expected behavior of a pronounced off-nucleus maximum, since they both stem from the wave function of the 2p orbiting electron, $\Psi_{21\pm 1}\left(r\right)$, relation (51) [45,46]. On the other hand, the obtained $U_{in}\left(r\right)$ seems to behave absolutely classically, at least at first sight. However, our simulations hint that even $U_{in}\left(r\right)$ somehow preserves a quantum mechanical trademark, as revealed by the off-nucleus, extremely weak, however discernible, maximum documented in Figure 6b. We argue that the $U_{in}\left(r\right)$ of relation (59) obtained with our methodology is exact and reliable. For instance, in [37] (see page 165, problem 4.7), the scalar potential, $U\left(r\right)$, was sought for the exact same 2p state of the electron in the hydrogen atom. A comparison between the two results, the one obtained in [37] by standard means and the one obtained here by using the expansion of $U\left(r\right)$ in the middle space, relations (35)–(37), documents their consistency.

The above examples indicate that our method could be of interest in atomic, molecular, and polymer physics. Indeed, starting from a so-called ‘trial’ wave function, $\Psi_{nlm}\left(r\right)$, the respective ‘trial’ electron charge density, $\rho_{nlm}\left(r\right)\sim {\left|\Psi_{nlm}\left(r\right)\right|}^{2}$, can be obtained. The latter can be used to calculate the accompanying ‘trial’ scalar potential, $U_{mid}\left(r\right)$, through relations (19), (33), and (34) (no azimuthal symmetry) or relations (35)–(37) (azimuthal symmetry). Then, $U_{mid}\left(r\right)$ can be implemented into the Schrödinger equation to seek an updated, more precise version of the wave function. The respective updated electron charge density will result in an updated scalar potential as well. This repetitive procedure can continue based on some criteria, e.g., until all involved physical entities converge at some steady form or until at least one of the involved physical entities exhibits the necessary quantitative agreement with information coming from experiments. On the other hand, experimental information of indisputable accuracy could accelerate the process. For instance, the electron charge density, as spatially distributed around the atomic nucleus, can be acquired based on high-resolution X-ray diffraction data and the recruitment of appropriate computational models (spherical, non-spherical/multipole distributions, etc.) [43,44,45]. With this information at hand, the accompanying scalar potential can be obtained through relations (35)–(37). Then, inserting the information of the experimentally accessed electron charge density and the theoretically calculated scalar potential into the Schrödinger equation, the parent wave function can ultimately be obtained.

### 7.2. Scalar Potential Obtained Under Screening Conditions (Thomas–Fermi Screening)

This case refers to the screening experienced by a fixed charge embedded in a solid medium consisting of charges of the opposite sign that are free to move (i.e., a nucleus placed in a reservoir of conduction electrons). The latter are attracted by the former so that they screen, at least partially, its electric scalar potential/vector field experienced by some other test charge. For instance, this case refers to the so-called Thomas–Fermi screening of the scalar potential/vector field of the positive nucleus by the surrounding negative electrons, usually discussed in solid state physics [48,49,50,51,52]. In general, the Thomas–Fermi screening is a nonlinear process, relatively difficult to tackle mathematically [50,51,52]. For the sake of the presentation, below, we consider the linearized case [48,49].

Briefly, we consider a localized, positive point charge, Q (proton of the nucleus), embedded in a reservoir of itinerant, negative point charges (free electrons). The unscreened potential of the ‘bare’ Q is simply given by the Coulomb law:

(60) $$U_{unsc}\left(r\right)=\frac{Q}{4\pi \varepsilon_{0}}\frac{1}{r}$$

**Figure 9:**
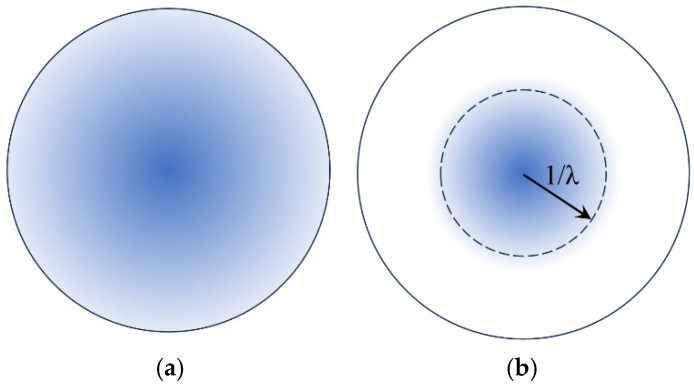
Illustrated schemes of the electric scalar potential of a nucleus in the (**a**) unscreened, $U_{unsc}\left(r\right)$, and (**b**) screened, $U_{sc}\left(r\right)$, states (both have polar and azimuthal symmetry). The screened state is due to the attraction of free electrons, of the surrounding reservoir, by the proton of the fixed nucleus. The dashed circle determines the screening radius, 1/λ, in respect to the former, unscreened state presented by the solid circle.

The screened potential is the total one defined by the superposition of the unscreened potential of the proton of the fixed nucleus and of an extra component originating from the attracted free electrons that surround the nucleus (now the proton can be viewed as ‘dressed’). It can be shown that the screened potential resembles the Yukawa one and is given by the following relation [48,49]:

(61) $$U_{sc}\left(r\right)=\frac{Q}{4\pi \varepsilon_{0}}\frac{1}{r}e^{-\lambda r}$$

where λ defines the respective screening radius, 1/λ. The situation is presented schematically in Figure 9 below, where panel (a) refers to the unscreened state, $U_{unsc}\left(r\right)$, while panel (b) to the screened one, $U_{sc}\left(r\right)$. In panel (b), we also present the former unscreened radius (solid circle) to highlight the significantly reduced screening radius, 1/λ. Thus, the screening of the proton by the surrounding electrons results in a significant reduction in the range of its scalar potential.

In this simple case, the total charge density, $\rho_{tot}\left(r\right)$, has only radial dependence, $\rho_{tot}\left(r\right)$, due to the isotropic nature of the former constituents, and is given by the following relation:

(62) $$\rho_{tot}\left(r\right)=\frac{Q}{4\pi }\left(4\pi \delta \left(r\right)-\lambda^{2}\frac{e^{-\lambda r}}{r}\right)$$

We use relations (35)–(37), where now l=0 should be inserted, to calculate the respective electric scalar potential. By using relations (36) and (37), after simple algebra, we obtain the following coefficient functions:

(63) $$A_{0,in}\left(r\right)=-\frac{Q}{4\pi \varepsilon_{0}}\lambda e^{-\lambda r}$$

and

(64) $$B_{0,out}\left(r\right)=\frac{Q}{4\pi \varepsilon_{0}}(1+\lambda r)e^{-\lambda r}$$

Thus, substituting relations (63) and (64) into relation (35), we obtain the desired electric scalar potential through the following relation:

(65) $$U_{in}\left(r\right)=(A_{0,in}\left(r\right)r^{0}+B_{0,out}\left(r\right)r^{-1})P_{l}(\cos\theta )=\frac{Q}{4\pi \varepsilon_{0}}\frac{1}{r}e^{-\lambda r}$$

in agreement with relation (61).

### 7.3. Scalar Potential Obtained from a Volume Density of Bound Charge or Pseudocharge, Originating from a Permanent Polarization

In this case, we deal with a dielectric and magnetic material in the form of a sphere (radius a) or a spherical shell (inner/outer radius, a/b, and thickness, c=b−a), having some kind of permanent polarization, P(r) and M(r), respectively. The relevant volume density of bound charges, $\rho_{b}(r)$, and pseudocharges, $\rho_{b,m}(r)$, is given through relations (38) and (39), respectively. Since in all the above cases we referred to electric charges, here we discuss two representative cases of magnetic pseudocharges originating from the permanent polarization of solid spheres and hollow spherical shells. Such toy models are usually found in important applications. For instance, in biomedicine, solid magnetic spheres are employed for diagnostic purposes, e.g., as contrast agents in magnetic resonance imaging, while hollow spherical magnetic shells are used for therapeutic aims, e.g., as containers for targeted drug delivery (see below).

#### 7.3.1. Sphere of Permanent Magnet with Radially Constant Polarization

Consider a sphere of radius a of a magnetic material with permanent polarization, $M\left(r\right)=M_{0}\hat{r}$. The relevant volume density of bound magnetic pseudocharges, $\rho_{b,m}\left(r\right)$, can be found by using relation (39). With simple algebra, we obtain the following:

(66) $$\rho_{b,m}\left(r\right)=-\frac{2M_{0}}{r}$$

**Figure 10:**
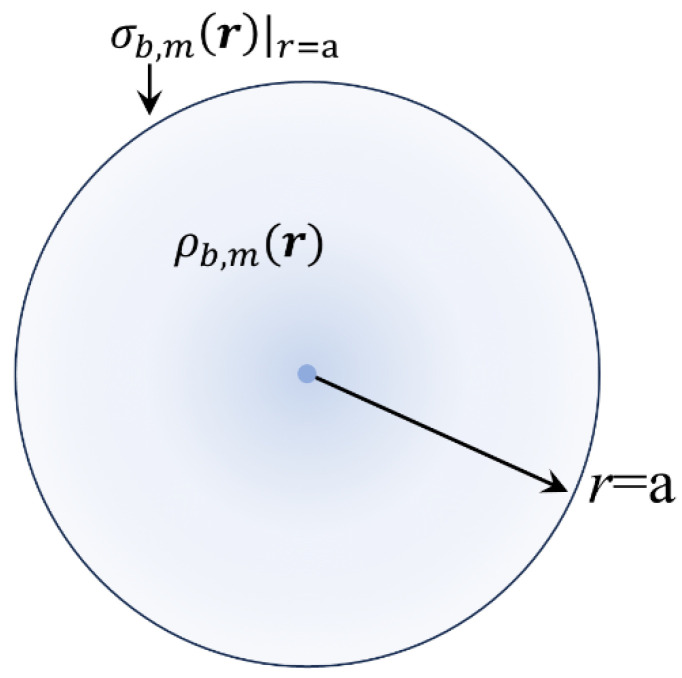
Illustrated scheme of the volume density of bound magnetic pseudocharges, $\rho_{b,m}\left(r\right)$, inherent to a magnetic sphere with radius a, of permanent polarization $M\left(r\right)=M_{0}\hat{r}$. The solid dot at the center of the sphere represents an infinite value of $\rho_{b,m}\left(0\right)$, accompanied by an abrupt fall across the radial direction evidenced by the color gradient; see relation (66). The surface density of bound magnetic pseudocharges, $\sigma_{b,m}\left(r\right){|}_{r=a}$, due to the discontinuity of $M\left(r\right)$ at the interface r=a is indicated as well.

A schematic illustration of $\rho_{b,m}\left(r\right)$ is shown in Figure 10 below.

We use relations (43)–(45) to calculate the respective magnetic scalar pseudopotential. Since the pseudocharge density, $\rho_{b,m}\left(r\right)$, depends solely on r, we should use l=0 in relations (43)–(45). Inserting the above information in relations (44) and (45), after simple algebra, we obtain the following coefficient functions:

(67) $$A_{0,in,m}\left(r\right)=-2M_{0}(a-r)$$

and

(68) $$B_{0,out,m}\left(r\right)=-M_{0}r^{2}$$

Thus, through relation (43), the desired magnetic scalar pseudopotential at the interior of the magnetic sphere is immediately obtained:

(69) $$U_{in,m}\left(r\right)=M_{0}(r-2a)$$

Interestingly, the respective magnetic field, $H_{in}(r)$, given through $H_{in}\left(r\right)=-\nabla U_{in,m}\left(r\right)$, becomes the following:

(70) $$H_{in}\left(r\right)=-M_{0}\hat{r}$$

so that at the interior of the sphere, the magnetic field $B_{in}\left(r\right)$ is $B_{in}\left(r\right)=\mu_{0}(H_{in}\left(r\right)+M\left(r\right))=0$. This implies a perfect depolarization process originating from the magnetic pseudocharges that reside at the volume ($\rho_{b,m}\left(r\right)=-2M_{0}/r$) and the surface ($\sigma_{b,m}\left(r\right){|}_{r=a}=(M\left(r\right)\cdot \hat{r}){|}_{r=a}=M_{0}$) of the sphere. Finally, it can be easily shown that the total magnetic pseudocharge (inside the volume 0≤r≤a and at the surface r=a) is zero, as it should be.

#### 7.3.2. Spherical Shell of Permanent Magnet with Radially Increasing Polarization

Now, consider a spherical shell of inner/outer radius a/b, that is, of thickness c=b−a, of a magnetic material with permanent polarization, $M\left(r\right)=M_{0}r/c=M_{0}(r/c)\hat{r}$. For instance, this property can originate from the varying character of the mass density of the parent material. The relevant volume density of bound magnetic pseudocharges, given through relation (39), is found to be as follows:

(71) $$\rho_{b,m}\left(r\right)=-\frac{3M_{0}}{c}$$

**Figure 11:**
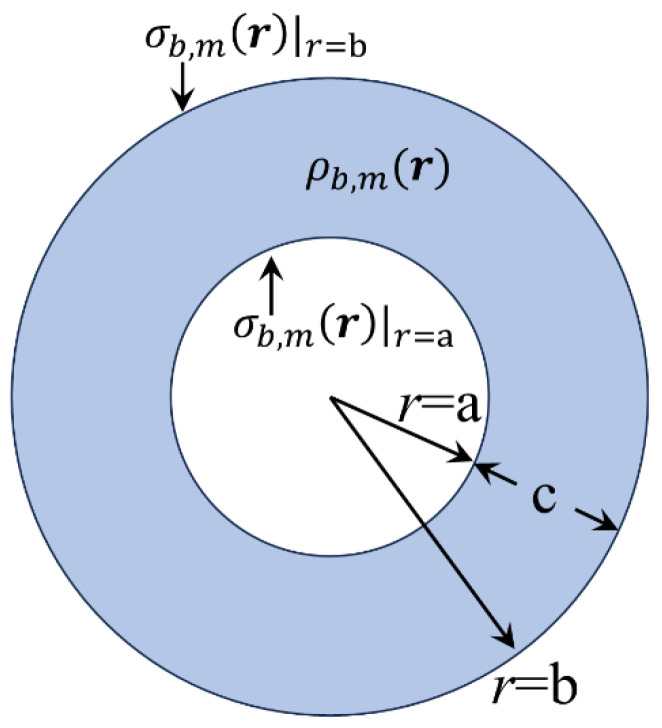
Illustrated scheme of the volume density of bound magnetic pseudocharges, $\rho_{b,m}\left(r\right)$, inherent to a magnetic spherical shell with inner/outer radius a/b, of permanent polarization $M\left(r\right)=M_{0}(r/c)\hat{r}$, where c=b−a is its thickness. The uniform color indicates that $\rho_{b,m}\left(r\right)$ is constant in the entire volume of the shell; see relation (71). The surface densities of bound magnetic pseudocharges, $\sigma_{b,m}\left(r\right){|}_{r=a}$ and $\sigma_{b,m}\left(r\right){|}_{r=b}$, due to the discontinuity of $M\left(r\right)$ at the interfaces r=a and r=b are indicated as well.

A qualitative schematic illustration of $\rho_{b,m}\left(r\right)$ is shown in Figure 11, below.

Now, $\rho_{b,m}\left(r\right)$ is constant. Thus, in relations (43)–(45), we should use l=0 to calculate the magnetic scalar pseudopotential. Inserting the above information in relations (44) and (45), after simple algebra, we obtain the following coefficient functions:

(72) $$A_{0,in,m}\left(r\right)=-\frac{3M_{0}}{c}\frac{b^{2}-r^{2}}{2}$$

and

(73) $$B_{0,out,m}\left(r\right)=-\frac{3M_{0}}{c}\frac{r^{3}-a^{3}}{3}$$

Thus, through relation (43), the desired magnetic scalar pseudopotential at the interior of the magnetic spherical shell is effortlessly obtained:

(74) $$U_{in,m}\left(r\right)=\frac{3M_{0}}{c}\left(-\frac{b^{2}}{2}+\frac{a^{3}}{3r}+\frac{r^{2}}{6}\right)$$

The respective magnetic field, H(r), at the interior of the magnetic spherical shell, given by $H_{in}\left(r\right)=-\nabla U_{in,m}\left(r\right)$, reads as follows:

(75) $$H_{in}\left(r\right)=\frac{3M_{0}}{c}\left(\frac{a^{3}}{3r}-\frac{r}{3}\right)\hat{r}$$

The respective magnetic field, $B\left(r\right)$, is given through the relation $B_{in}\left(r\right)=\mu_{0}(H_{in}\left(r\right)+M\left(r\right))=\mu_{0}(3M_{0}/c)(a^{3}/3)(\hat{r}/r^{2})$. Notice that $\nabla \cdot B_{in}\left(r\right)=\mu_{0}(3M_{0}/c)(a^{3}/3)\nabla \cdot (\hat{r}/r^{2})$, while by taking into account the identity $\nabla \cdot \left(\hat{r}/r^{2}\right)=4\pi \delta (r)$, we easily see that $\nabla \cdot B_{in}\left(r\right)=0$ since the center of the spherical shell is not included within the range 0<a≤r≤b. Thus, the solutions of $U_{in,m}\left(r\right)$, $H_{in}\left(r\right),$ and $B_{in}\left(r\right)$ obtained by using our expressions are well defined on physical grounds. Finally, it can be easily shown that the total magnetic pseudocharge (inside the volume a≤r≤b and at the two surfaces r=a and r=b) is zero, as it should be.

### 7.4. Applicability of Our Approach in Other Areas of Science and Engineering

Our theoretical approach to the multipole expansion and the derivation of the solution of the Poisson equation can be quite useful in other areas of science, such as soft and biological matter, and engineering, such as scattering and biomedical. For instance, dielectric spherical structures are routinely used in scattering applications and invisibility cloaks [53,54], to model cells in suspensions [55,56], and in the process of dielectrophoresis, e.g., the manipulation of cells and colloidal particles subjected to an external electric field [57,58,59]. Also, magnetic spherical structures, solid spheres and hollow cells, are commonly employed in biomedicine, namely in magnetophoresis, e.g., magnetic and immunomagnetic sorting, and in the manipulation/delivery of cells [60,61,62,63,64], in diagnostic radiology, e.g., in magnetic resonance imaging, Positron Emission Tomography, and Single Photon Emission Computed Tomography [65,66,67,68], and in therapeutic ones, e.g., blood purification [69,70,71,72] and hyperthermia [73,74]. More information on these issues can be found in [75].

Finally, except for the case of the magnetic materials discussed above, our theoretical model could be used to assess the magnetic polarization, i.e., magnetization, M(r), of superconductors (see [76,77,78,79] and references therein). In principle, our approach can be applicable in various ways in this field. For instance, the magnetic polarization of superconductors, M(r), depends on the magnetic field. M(r) can be measured experimentally through DC and AC susceptibility techniques, either globally (e.g., via SQUID and AC magnetometry [77]) or locally (e.g., via Hall magnetometry [80]). Thus, from the experimental information on $M\left(r\right),$ we can model/calculate the volume and surface densities of magnetic pseudocharges, $\rho_{b,m}\left(r\right)=-\nabla \cdot M(r)$ and $\sigma_{b,m}\left(r\right)=M(r)\cdot \hat{n}$, respectively (taking into account whether the superconductor is in the Meissner or mixed state as well). From these densities, we can calculate the magnetic pseudopotential, $U_{m}(r)$, based on our method, relations (40)–(45). Then, the magnetic field can be obtained through $H\left(r\right)=-\nabla \cdot U_{m}(r)$. The result should be consistent with the starting hypothesis. The above interesting suggestion needs clarification in future work.

## 8. Conclusions

Though in the literature the multipole expansion of the scalar potential, $U\left(r\right)$, is reproduced only away from any source, that is, in the inside space, $U_{in}\left(r\right)$, and outside space, $U_{out}\left(r\right)$, here, we investigated this issue in the middle space wherein a volume source resides, for both dielectric and magnetic materials. We introduced a consistent expression for $U_{mid}\left(r\right)$, which among others, (i) satisfies the continuity at both interfaces, with the inside space, $U_{mid}\left(r\right)=U_{in}\left(r\right)$, and the outside space, $U_{mid}\left(r\right)=U_{out}\left(r\right)$, and (ii) satisfies the Poisson equation. The expression of $U_{mid}\left(r\right)$ was derived both heuristically by using arguments based on the underlying physics and purely mathematically by means of the method of variation of parameters. The latter derivation is a standalone, quite interesting finding that uncovers the mathematical origin of the so-called multipole expansion of the scalar potential, $U\left(r\right)$, in spaces wherein sources reside. Indeed, the generic expression of $U_{mid}\left(r\right)$ introduced here is an effective solver of the Poisson equation, as was documented for a number of representative cases found in the literature. Finally, the present work investigated the static limit, i.e., a DC scalar potential/vector field, while obviously applying the quasi-static limit, i.e., an AC scalar potential/vector field of low frequency. It can be easily generalized to describe the fully dynamic case, thus being useful in time-dependent applications of both homogeneous and nonhomogeneous, dielectric and magnetic materials.

## Data Availability

The original contributions presented in the study are included in the article, further inquiries can be directed to the corresponding author.
